# Gaze direction biases emotion categorisation in schizophrenia

**DOI:** 10.1016/j.scog.2020.100181

**Published:** 2020-05-21

**Authors:** Nathan Caruana, Christine Inkley, Marwa El Zein

**Affiliations:** a Perception in Action Research Centre, Macquarie University, Sydney, Australia; b Department of Cognitive Science, Macquarie University, Sydney, Australia; c Institute of Cognitive Neuroscience, University College London, United Kingdom

**Keywords:** Face processing, Emotion, Gaze, Social perception, Computational modelling, Schizophrenia

## Abstract

The successful integration of eye gaze direction and emotion cues from faces is important not only for co-ordinated interactions, but also for the detection of social signals alerting us to threat posed by a conspecific, or elsewhere in our immediate environment. It is now well-established that people with schizophrenia experience aberrant eye gaze and facial emotion processing. These social-cognitive differences might contribute to the maintenance of socially-themed delusions which are characterised by the hyper-attribution of threatening intentions to others. However, no study has directly examined whether the mechanisms which govern the integration of eye gaze and emotion information diverge in schizophrenia, and more importantly, whether this reflects a fundamental ‘bottom-up’ perceptual deficit or a ‘top-down’ cognitive bias. Fifteen outpatients diagnosed with schizophrenia and 21 healthy age- and IQ-matched controls performed an emotion categorisation task (anger/fear) on morphed facial expressions of anger or fear, displaying either direct or averted gaze. Results in both controls and patients replicated the previous finding that combinations of anger with direct gaze, and fear with averted gaze – which signal a relevant threat to the observer – benefited from more accurate emotion recognition than alternate gaze-emotion combinations. Bayesian model selection revealed that for patients this effect was mediated by a shift in decision bias towards emotions which signal self-relevant threat, rather than a change in sensitivity as observed in controls. These results critically highlight a different cognitive mechanism governing gaze and face-cued emotion integration in schizophrenia, which has a top-down influence on the evaluation of perceptual input.

## 1 Introduction

Schizophrenia is a psychiatric condition that affects multiple cognitive processes – including social and affective cognition – which can negatively impact social functioning and quality of life (Kee et al., 2003; Penn et al., 1996). It is well established that the processing of social information conveyed by facial expressions and eye gaze is divergent in schizophrenia (see Billeke and Aboitiz, 2013 for review). In particular, meta analyses estimate large overall effect sizes for face-related emotion processing deficits in schizophrenia compared to healthy controls (e.g., d = −0.91, d = −0.85, and g = 0.89 respectively; Chan et al., 2010; Kohler et al., 2010; Savla et al., 2013). However, studies of face perception have been unable to directly test whether these differences in schizophrenia – and poor social cognition more broadly – stem from a fundamental, ‘bottom-up’ aberration in the perception of social stimuli (e.g., Darke et al., 2013; Doop and Park, 2009; Fletcher and Frith, 2009; Silverstein, 2016; Uhlhaas and Mishara, 2006) or a cognitive bias which has a ‘top-down’ influence on the conscious evaluation of perceptual information. Recent work on the psychometric assessment of social cognition in schizophrenia highlights the need to make this distinction between cognitive *deficits* and *biases* clearer (Pinkham et al., 2016b; Roberts and Pinkham, 2013; Walss-Bass et al., 2013). Such a distinction is key to achieving more specific explanations of divergent social functioning in schizophrenia.

Despite the dearth of direct empirical evidence, there are several streams of *indirect* evidence which suggest a top-down cognitive bias in schizophrenia, rather than a fundamental perceptual deficit, drives the divergent processing of social cues in faces. First, whilst there is very substantial evidence of face processing deficits in schizophrenia (see above) this is based on a body of literature in which experimental studies have required patients to make conscious face judgements. However, recent studies using continuous flash suppression (CFS) tasks – which temporarily supress visual stimuli from conscious awareness, and do not require participants to make stimulus judgments – have shown that patients, like healthy controls, demonstrate intact visual prioritisation of faces over non-face stimuli (Caruana et al., 2019c) and faces with direct gaze over averted gaze (Seymour et al., 2016). As such, these studies suggest that a top-down cognitive bias might offer a more likely explanation for deficits observed in conscious face evaluation tasks.

A second stream of studies suggests that patients are more likely to detect intentionality and threat in the social signals conveyed by others (e.g., Blakemore et al., 2003; Haggard et al., 2003). Furthermore, impairments of emotion categorisation from faces seem to be exacerbated in schizophrenia for faces with threat-relevant emotions (e.g., anger, fear, disgust and sadness; also see Bediou et al., 2005; Brüne, 2005; Comparelli et al., 2014; Edwards et al., 2001; Kohler et al., 2003; Pinkham et al., 2003). Indeed, patients in general have an increased tendency to over-attribute meaning to irrelevant or ambiguous stimuli (Phillips et al., 2003). Together, this suggests that the potential for threat to be signalled by face stimuli may drive differences in the evaluation of these faces in schizophrenia, rather than a reduced capacity to process the configural features of faces which convey expressed emotions. In line with such an interpretation, actively-paranoid patients have been found to (1) be more likely to mis-categorise neutral faces as angry (Pinkham et al., 2011); (2) be more likely to mis-categorise neutral words as unpleasant (Holt et al., 2006); and (3) demonstrate more profound social-cognitive biases and poorer social functioning than non-paranoid patients (Pinkham et al., 2016a). Specifically, on standardised social-cognitive tasks, paranoid patients are more likely to interpret the intentions of others in ambiguous social situations as hostile, and to evaluate ambiguous faces as untrustworthy. Critically, this study revealed that differences were only seen on psychometric tasks that provided measures of social-cognitive ‘bias’ rather than ‘capacity’ (see Roberts and Pinkham, 2013; Walss-Bass et al., 2013 for a broader discussion on this distinction). Together, these findings indicate an over-attribution of threat to ambiguous stimuli in schizophrenia, particularly in those who are actively experiencing paranoid delusions. It is therefore possible that these biases result from the long-term maintenance of persecutory delusions in which others are believed to be untrustworthy with intentions to inflict harm.

Finally, evidence for cognitive biases influencing social information processing comes from studies of gaze processing in schizophrenia. Much like the face-cued emotion processing literature, gaze processing studies in schizophrenia have largely examined the *conscious* evaluation of eye gaze direction. For instance, gaze-cueing studies have shown that patients with schizophrenia exhibit an enhanced gaze congruency advantage (e.g., Langdon et al., 2006; Langdon et al., 2017). Under conditions of brief stimulus presentation, patients have also been more likely than healthy controls to claim that averted gaze is directed at them (Hooker and Park, 2005; Rosse et al., 1994; Tso et al., 2012). This ‘direct gaze bias’ has been shown to persist even when the eyes were edited out of the face stimulus entirely, once again suggesting a top-down cognitive bias, rather than aberrant perceptual processing (Hooker and Park, 2005). Further, studies that do not involve a self-referential judgement (e.g., “*Are the eyes looking at you*?”), but simply instruct participants to report whether the eyes are averted to the left or right, have failed to reliably identify a direct gaze bias in patients. Together, these findings again suggest that aberrant gaze processing in schizophrenia likely reflects an evaluative, self-referential bias rather than deficits in visual perception (Franck et al., 1998, Franck et al., 2002; Seymour et al., 2017). Yet, despite these converging streams of evidence – which support the view that a cognitive bias mechanism drives aberrations in the evaluation of emotions from faces in schizophrenia – no study has provided a paradigm which can directly test this claim.

### 1.1 Examining the interactions of gaze direction and emotion cues

Promise for such a paradigm comes from recent work examining the integration of gaze direction and emotion cues in faces. In healthy adults, it is now well-established that humans use gaze direction cues to facilitate the categorisation of angry and fearful expressions which can signal threat to an observer (e.g., Adams Jr and Kleck, 2003, Adams Jr and Kleck, 2005; Cristinzio et al., 2009; El Zein et al., 2015b; N'diaye et al., 2009; Sander et al., 2007). This is because gaze direction contextualises the locus of the potential threat. Specifically, **angry faces with direct gaze** may signal that the observed person intends to harm the observer. Alternatively, a **fearful face with averted gaze** may signal an external but proximal source of danger (e.g., a predator) which may pose a relevant threat to both the observer and the observed (Sander et al., 2007). Recently, the mechanism underlying the increased recognition of these self-relevant gaze and emotion combinations (hereafter referred to as “Threat+” faces) was established in a signal detection theory framework (El Zein et al., 2015b; Ioannou et al., 2017). In this framework, there are two competing models which explain the underlying mechanism driving this contextual influence of gaze direction on emotion recognition: (1) a perceptual sensitivity mechanism or (2) a decision bias mechanism.

#### 1.1.1 Perceptual sensitivity

The perceptual sensitivity model in this framework assumes that gaze direction increases the observer's sensitivity to the perceptual features that allow them to categorise a face as angry or fearful. As such, angry faces with direct gaze would be perceived as more angry – and fearful faces with averted gaze as more fearful – than alternate gaze-emotion combinations (i.e., anger-averted, fear-direct; hereafter referred to as “Threat−” faces). This sharpening of perceptual sensitivity would correspond to a boost in the bottom-up perceptual processing of Threat+ faces, especially when faces show anger and fear emotions at very low intensities (but not for neutral faces), which in turn would enable observers to more readily and accurately categorise the emotion conveyed by the face (El Zein et al., 2015a, El Zein et al., 2015b, Ioannou et al., 2017). Using Bayesian model selection methods, previous studies have now consistently demonstrated that this perceptual sensitivity model best accounts for the increased emotion categorisation accuracy for Threat+ over Threat− faces in healthy adults and adolescents (El Zein et al., 2015a, El Zein et al., 2015b; Ioannou et al., 2017).

#### 1.1.2 Decision bias

Alternatively, the decision bias model assumes that gaze direction cues bias the observer's decision about whether a face is angry or fearful in the direction of the combination signalling a higher threat. Maximal effects of this bias would be observed for neutral face expressions, in which direct gaze will bias observers to categorise the face as angry and neutral faces with averted gaze as fearful. This shift in decision bias – which also impacts on the categorisation of neutral faces with no perceptual emotion cues – would correspond to a top-down contextual influence of gaze cues on the recognition of emotional expression.

### 1.2 Current study

The current study aimed to adopt El Zein et al., 2015a, El Zein et al., 2015b emotion categorisation paradigm to investigate whether patients – like healthy adults – exhibit an increased perceptual sensitivity for Threat+ faces, or rather, whether gaze direction cues bias the evaluation of faces as expressing the emotion that would convey a self-relevant threat. In line with the latter, we anticipated that emotion categorisation performance in patients would be best characterised by a decision bias model. This means that even for neutral faces, patients would exhibit a decision bias towards anger when presented with direct gaze, and a bias towards fear for averted gaze. Contrastingly, we expected – as has been consistently demonstrated in past studies – that categorisation performance in healthy controls would be better characterised by a perceptual sensitivity model with more accurate categorisations of Threat+ combinations, even when the intensity of emotion cues are low. Together, such findings would directly evidence a top-down cognitive bias mechanism in schizophrenia which influences the appraisal of visual social cues as signals for self-directed threat.

## 2 Method

### 2.1 Ethical statement

This study was approved by the Human Research Ethics Committee at Macquarie University (MQ; reference number: 5201200021). Participants received payment for their time and provided written consent before participating.

### 2.2 Participants

Twenty outpatients diagnosed with Schizophrenia or Schizoaffective Disorder (10M/10F) and 21 healthy controls (14M/7F) participated in this study. To classify for inclusion in the study participants could not have had any current or past neurological disease or injury resulting in a concussion or being unconscious for more than 1 h. All participants were additionally screened for any history of substance abuse (as per DSM-V criteria) and were required to have a minimum eight years of formal education. Finally, all participants had normal or corrected-to-normal vision.

Five patients were excluded from analyses because they failed to accurately categorise emotions on at least 60% of trials, resulting in a final sample of 15 patients (6 M, 9F). This ensured adequate individual data to reliably characterise the mechanisms underlying the influence of gaze direction on emotion categorisation. Groups did not differ significantly on age (Patients *M* = 53.93, *SD* = 8.37; Control *M* = 48.14, *SD* = 13.47; *t*(34) = −1.47, *p* = 0.150, BF_10_ = 0.746). The National Adult Reading Test was administered as a measure of premorbid intelligence (NART; Nelson and Willison, 1991). Again, groups did not significantly differ on the NART full-scale IQ estimate (Patients *M* = 106.47, *SD* = 9.61; Control *M* = 106.53, *SD* = 9.89; *t*(34) = 0.02, *p* = 0.986, BF_10_ = 0.325).

Patients were recruited from the Macquarie University Belief Formation Volunteer Register. All patients were diagnosed by a clinical psychologist or psychiatrist before being recruited into the study and were on a stable dose of antipsychotic medication. Participants were also assessed against current DSM-V criteria using the Diagnostic Interview for Psychosis (Castle et al., 2006). Symptom severity was assessed using the Scales for Assessment of Positive and Negative Symptoms (SAPS & SANS; Andreasen, 1983, Andreasen, 1984). This revealed that the patients in our sample were stable with mild symptomology (see Table 1).

**Table 1:** Symptom ratings for patients on the SAPS and SANS.

	M	SD	Range
Negative symptoms (SANS)			
Affective flattening or blunting	1.90	1.17	0–4
Alogia	0.95	1.10	0–3
Apathy	2.75	1.12	0–4
Anhedonia	3.00	1.26	0–5
Attention	1.05	1.10	0–3
Positive symptoms (SAPS)^a^			
Hallucinations	1.60	1.50	0–4
Delusions	1.90	1.45	0–4
Bizarre behaviour	0.80	1.01	0–2
Positive thought disorder	1.65	1.23	0–4

Note. Ratings on the SAPS and SANS are provided on a 5-point scale; 0 = not present; 1 = questionable; 2 = mild; 3 = moderate; 4 = marked; 5 = severe.

We used a structured interview to screen controls. This interview was based on the affective, psychotic and substance abuse screening modules from the Structural Clinical Interview for Axis 1 Disorders previously outlined under DSM-IV (SCID-1; First et al., 2002). This interview allowed us to screen for and exclude controls who had a psychiatric diagnosis of any kind, including but not limited to schizophrenia. Control participants also completed the brief version of the Schizotypal Personality Questionnaire (SPQ—B; Raine and Benishay, 1995). The range of obtained scores (*M* = 4.38, *SD* = 3.67) were consistent with previous studies involving non-clinical community samples (e.g., Compton et al., 2007).

### 2.3 Apparatus and stimuli

**Fig. 1:**
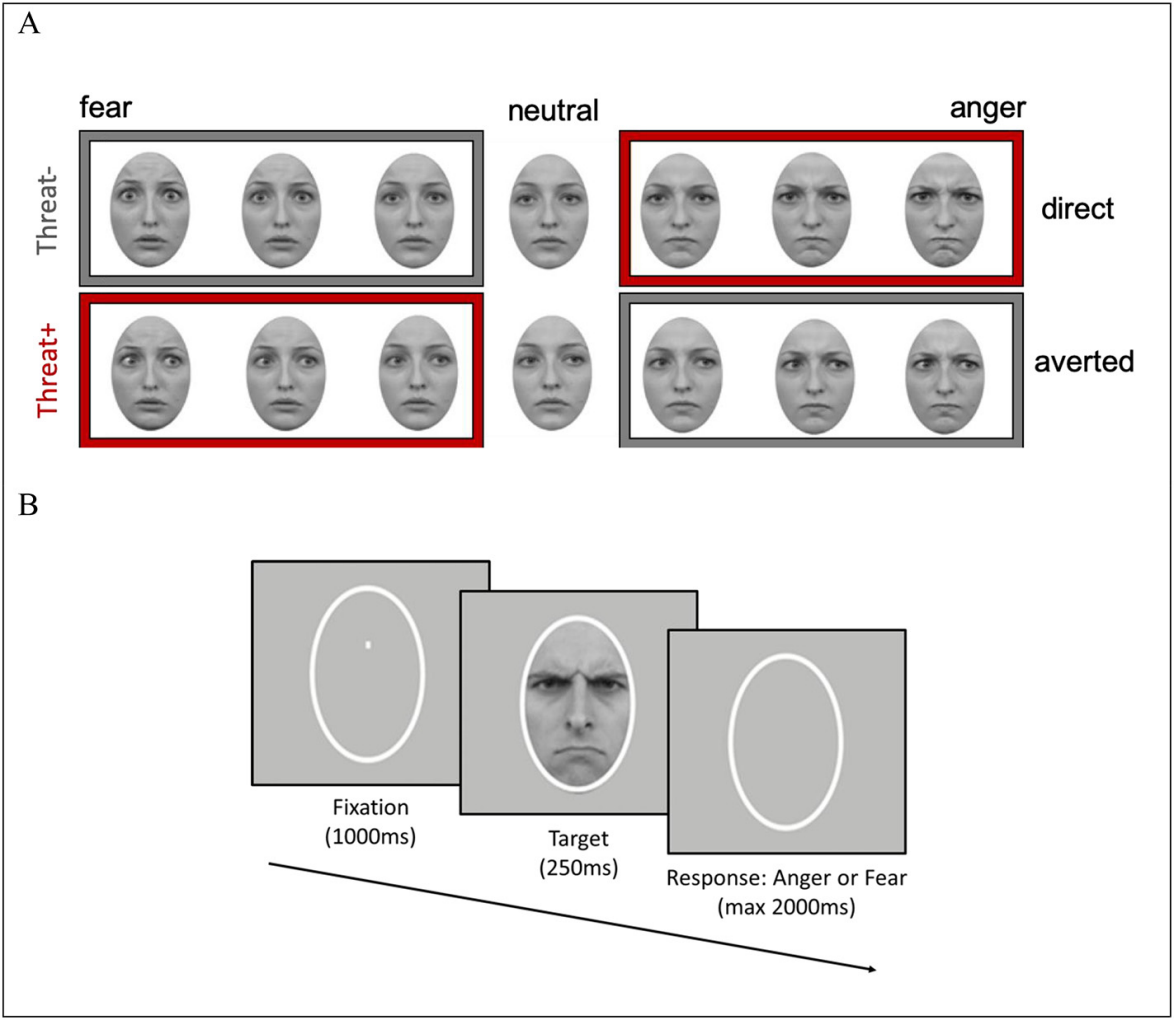
(A) Stimuli and (B) trial sequence for emotion categorisation task. Threat+ faces (in red frames) comprised anger and direct gaze or fear and averted gaze combinations. Threat− faces (in grey frames) comprised anger and averted or fear and direct gaze combinations. Three levels of morphs are shown for each emotion as examples, however, the stimuli comprised of 7 levels of morphs,

The stimulus set comprised 20 identities (10 females) adapted from the Radboud Faces Database (for a detailed description of stimulus properties and development see; El Zein et al., 2015a, El Zein et al., 2015b; Langner et al., 2010) and have been used in several previous studies examining gaze-emotion integration (Caruana et al., 2019a; El Zein et al., 2015a, El Zein et al., 2015b; Ioannou et al., 2017). Within each identity, the face stimuli varied in emotion (neutral, angry or fearful) and gaze direction (directed at the participant or averted 45° to the left or right). Each face identity set comprised seven levels of emotion morphs from neutral to angry and from neutral to fearful. This resulted in 30 face stimuli per identity: [(7 levels of morphs ∗ 2 emotions ∗ 2 gaze directions = 28) + (2 neutral stimuli with direct and averted gaze)]. With 20 identity sets, this resulted in a total stimulus set of 600 faces (see Fig. 1A for an example). The morphing was previously calibrated between angry and fearful expressions such that the emotional expressions of anger and fear conveyed the same intensity of emotion expressions (see Caruana et al., 2019a; El Zein et al., 2015a, El Zein et al., 2015b for detailed accounts on the stimulus calibration method). All faces appeared as greyscale images and were cropped to remove any visible hair.

### 2.4 Procedure

Using the Psychophysics-3 Toolbox (Brainard, 1997; Pelli, 1997), stimuli were projected on a black screen. Each trial began with a white oval for 500 ms, followed by a white fixation point presented within the oval at participants' eye-level for 1000 ms. Following fixation, the face stimuli appeared within the oval for 250 ms, and then disappeared. The participants' task was to decide whether the faces expressed Anger or Fear by pressing one of two keyboard buttons with the left or right hand (Fig. 1B). An Anger/Fear response mapping was used (e.g., Anger: Left hand, Fear: Right hand) which was kept constant for each subject, but counterbalanced across subjects. All stimuli were presented once, resulting in a total of 600 trials. The experiment was divided into five blocks, each comprising 120 trials, that were balanced for emotion, gaze directions, gender and morph levels.

### 2.5 Analyses

A repeated-measures ANOVA was performed on the percentage of correct responses with gaze direction (direct, averted), emotion (anger, fear), and intensity (7 levels of morphs) as within-subjects factors, and group (patients, controls) as a between-subject factor. The same analysis was performed within each group independently. Student *t*-tests were conducted to directly compare performance between Threat+ and Threat− face conditions.

#### 2.5.1 Model selection

To characterise the mechanisms underlying the enhanced categorisation of Threat+ faces, we performed model-based analyses inspired by signal detection theory (see El Zein et al., 2015a, El Zein et al., 2015b; Ioannou et al., 2017). We compared between two models which assumed two different mechanisms accounting for an increased emotion categorisation accuracy for Threat+ over Threat− faces; (1) a change in perceptual sensitivity and (2) a change in decision bias.

##### 2.5.1.1 Model 1 – perceptual sensitivity model

In our previous studies conducted with healthy adults and adolescents, as well as adolescents with autism (El Zein et al., 2015a, El Zein et al., 2015b; Ioannou et al., 2017), a perceptual sensitivity model was found to best characterise the emotion categorisation data. In this model, gaze direction was found to selectively increase categorisation sensitivity to emotions signalling higher threat (i.e., Threat+ versus Threat− faces). This model assigns a different sensitivity to emotions in Threat+ versus Threat− conditions.

##### 2.5.1.2 Model 2 – decision bias model

Alternatively, the decision bias model assumes that gaze direction biases emotion recognition in favour of the interpretation signalling higher threat (i.e., anger for a direct gaze, fear for an averted gaze).

For our comparison between these two models, we used a Bayesian model selection method based on the model evidence (i.e., log-likelihood). This method allows us to quantify the support of one model over the other, by estimating which model best fits the observed data. As both compared models have the same number of parameters, we did not use any method to account for the complexity of the model (i.e., number of parameters). Fixed-effects and random-effects statistics are reported. The fixed-effect comparison assumes all the participants relied on the same mechanisms to generate their decisions. The random-effects comparison is more conservative and allows each participant to rely on different mechanisms in order to generate their decisions. For the former, we report the Bayes factor as the ratio of model evidence for the compared model (Jeffreys, 1961; Kass and Raftery, 1995). For the latter, we computed support for the winning model by the exceedance probability (p_exc_), which is the probability that participants' behaviour was governed by the model's assumed mechanism, compared to that of the alternative model. Once we identified the winning model for each group (controls and patients), we ran statistical analyses on the maximum-likelihood parameter estimates extracted from the winning model. We report the statistical analyses from the winning model in each group, and depict on the results figure below, only the parameter estimates extracted from these winning models.

## 3 Results

### 3.1 Performance

**Fig. 2:**
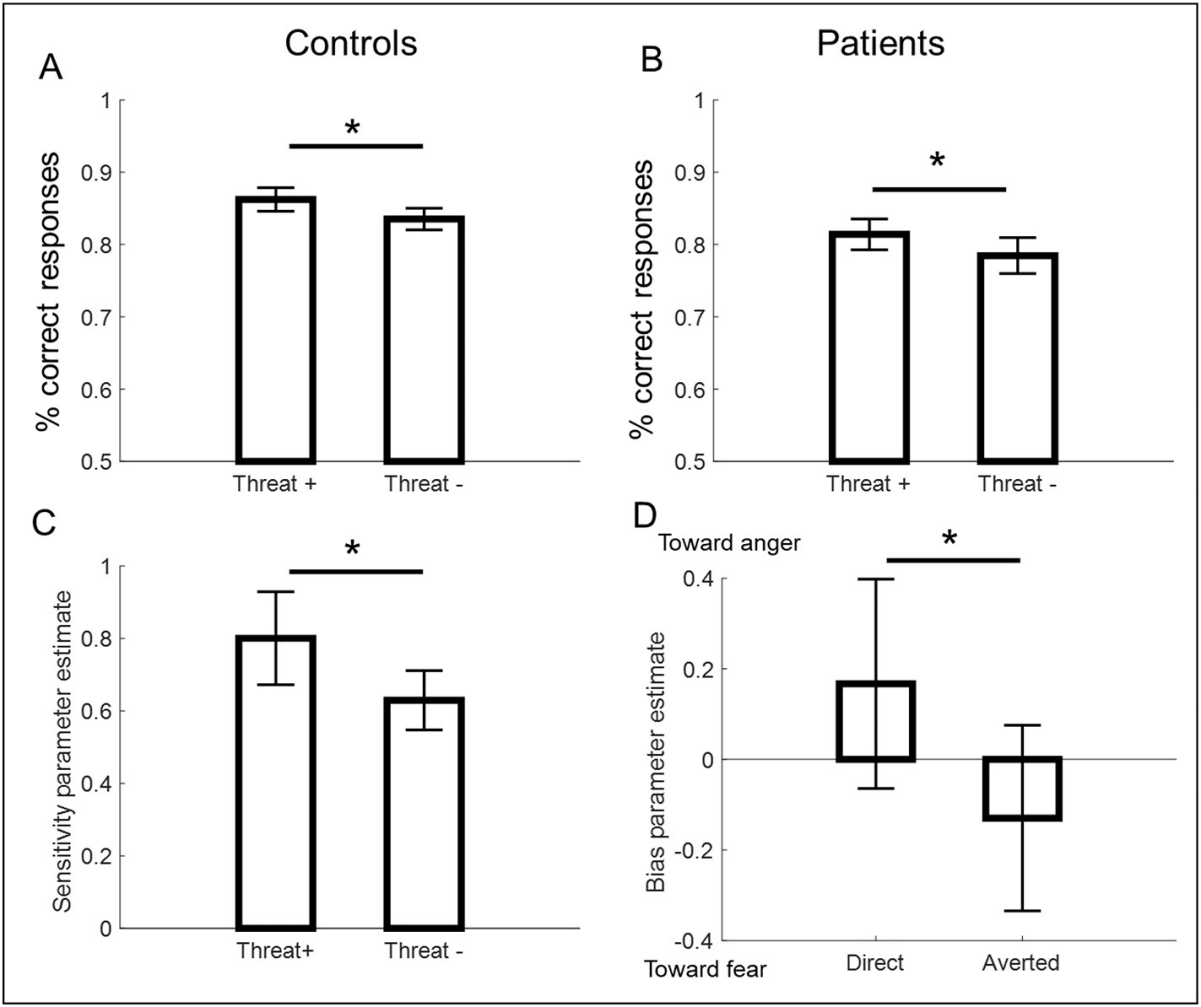
Emotion categorisation performance and model selection. (A) Percentage of correct responses for Threat+ and Threat− in controls (B) Percentage of correct responses for Threat+ and Threat− in patients. (C) Sensitivity parameter estimate for Threat+ and Threat− from the winning model in controls. (D) Bias parameter for direct and averted gaze from the winning model in patients. Positive values indicate a bias towards anger response, whilst negative values indicate a bias towards fear responses. *p < 0.05 (two tailed *t*-test in A–B, one-tailed *t*-test in C–D).

Emotion categorisation accuracy increased with the level of displayed emotions for both groups (*F*_(6,204)_ = 91.74, *p* < 0.001, *η*^*2*^ = 0.175). A main effect of emotion was also observed as anger was better categorised than fear (*F*_(1,34)_ = 4.19, *p* = 0.048, *η*^*2*^ = 0.023). Importantly – and in line with our predictions and previous literature – we found evidence for a significant emotion by gaze interaction (*F*_(1,34)_ = 11.61, *p* = 0.002, *η*^*2*^ = 0.008). This effect, however, did not interact with group (*F*_(1,34)_ = 0.0218 *p* = 0.893, *η*^*2*^ = 0.000), and was significant in both patients (*F*_(1,14)_ = 5.34, *p* = 0.037, *η*^*2*^ = 0.007) and controls (*F*_(1,20)_ = 6.46, *p* = 0.019, *η*^*2*^ = 0.009) when analysed separately. This emotion by gaze interaction was characterised by more accurate categorisation of Threat+ than Threat− faces in both controls (*t*_(20)_ = 2.26, *p* = 0.03, *d* = 0.494; see Fig. 2A) and patients (*t*_(14)_ = 2.66, *p* = 0.018, *d* = 0.688; see Fig. 2B).

#### 3.1.1 Model selection

In order to characterise the mechanisms underlying better performance for Threat+ than Threat− faces, we compared the perceptual sensitivity model (i.e., Model 1: testing for an effect of sensitivity to emotional expression intensity) and decision bias model (i.e., Model 2: testing for a bias towards one emotion over another). Our Bayesian model selection analysis revealed that Model 1 (perceptual sensitivity) explained the control group data better than Model 2 (decision bias shift; BF_10_ ≈ 10^2.22^, exceedance probability p_exc_ > 0.86). Maximum-likelihood estimates of the perceptual sensitivity parameter extracted from the winning model were significantly larger for Threat+ than Threat− faces (one tailed *t*-test, *t*_(20)_ = 1.93, *p* = 0.034, *d* = 0.423; Fig. 2C). This means that for controls, anger with direct gaze and fear with averted gaze benefited from an increased perceptual recognition as compared to the other gaze-emotion combinations.

Contrastingly, for the patient group, Model 2 (decision bias shift) explained the data better than Model 1 (perceptual sensitivity; BF_10_ ≈ 10^3.17^, p_exc_ > 0.84). Maximum-likelihood estimates of the bias parameter extracted from the winning model differed significantly for direct and averted gaze (one tailed *t*-test, *t*_(14)_ = 2.54, *p* = 0.01, *d* = 0.657; Fig. 2D). This reveals that for patients, direct gaze biased emotion categorisation responses towards ‘angry’, and averted gaze biased responses towards ‘fear’. We have also included comparisons with a third model in Supplementary material 1 which includes a possible effect of both perceptual sensitivity and decision bias. These additional model comparisons rule out the possibility that the increased accuracy for Threat+ over Threat− faces is explained by *both* a change in the sensitivity and bias parameter. Altogether, these results provide further evidence that the effects in each group were specific to a change in sensitivity for controls, and a change in decision bias for patients.

## 4 Discussion

The capacity to detect threat signalled by the faces of conspecifics is important for enabling humans to avoid threats posed by either other humans, or external sources of danger in the immediate environment (Sander et al., 2007). Recent research has revealed that humans integrate gaze direction cues when categorising face-cued emotions to increase their perceptual sensitivity for faces that potentially convey a self- and threat-relevant signal (El Zein et al., 2015a, El Zein et al., 2015b). This suggests that in healthy adults, gaze direction cues have a bottom-up influence on the evaluation of emotion expressions, such that fearful faces with averted gaze and angry faces with direct gaze (i.e., Threat+ faces) are categorised more accurately than alternative gaze-emotion combinations (i.e., Threat− faces). The current study aimed to examine whether this ability is supported by a different mechanism in schizophrenia, in line with a top-down cognitive bias account of social-cognitive difficulties.

We found that, like healthy controls, patients were more accurate in categorising Threat+ than Threat− faces. Critically, however, Bayesian modelling of the emotion categorisation data revealed that the underlying mechanism driving this effect differed in patients. Previous modelling of emotion categorisation data has revealed that this phenomenon in neurotypical humans is likely driven by a ‘perceptual sensitivity’ mechanism – whereby gaze direction acts as a contextual cue which increases one's ability to sensitively (i.e., more accurately) categorise emotions (El Zein et al., 2015a, El Zein et al., 2015b). This finding has been replicated several times now, in another study involving autistic and neurotypical adolescents (Ioannou et al., 2017), and again in the control participants of the current study. However, contrastingly, data from the patient group in the current study was best explained by a ‘decision bias’ model. This suggests, that in patients the increased accuracy for categorising Threat+ faces was driven by direct gaze *biasing* patients towards the categorisation of angry faces, and likewise, averted gaze *biasing* patients towards categorising fearful faces. This bias in patients is most clearly observed when categorising neutral faces, as they were more likely to categorise a neutral face as (1) angry when gaze was directed at the participant, and (2) fearful when gaze was averted. In other words, patients were biased to assume that the face displayed the emotion that would be most threatening to themselves given the gaze cue provided. This bias manifests for neutral faces even though they offer no visual evidence for either emotion. This direct evidence for a decision bias mechanism aligns with indirect supporting evidence from the emotion and gaze processing literature in schizophrenia, which until now have largely remained separate.

Previous studies revealed that patients with schizophrenia exhibit deficits in emotion categorisation from faces – particularly for negatively-expressed emotions – even when positive symptoms are mild and effectively controlled using medication (Kohler et al., 2010). In contrast, impairments in recognising identities from faces are not reliably observed in schizophrenia, suggesting that the perceptual processing of invariant face features is intact (see Darke et al., 2013; Watson, 2013 for relevant reviews). These discrepant findings may therefore reflect variation in the higher-level cognitive demands of the tasks used to assess face perception (Bortolon et al., 2015; Kohler et al., 2010). As such emotion processing impairments may reflect a primarily top-down cognitive bias which impacts the conscious evaluation of perceptual input – rather than an aberration of basic perceptual mechanisms. Indeed, this may reflect a downstream consequence of the mentalising impairments widely reported in patients with schizophrenia – which in turn may facilitate the maintenance of positive symptoms such as persecutory delusions (Brüne, 2005; Langdon et al., 2014; Sprong et al., 2007). In line with this view, actively-paranoid patients are also more likely to mis-categorise neutral faces as angry (Pinkham et al., 2011), which reflects a top-down bias to perceive a neutral stimulus as emotionally salient and threatening. Our modelling analyses also support this last finding, as they suggest that patients tend to perceive neutral stimuli as being more threat-relevant. However, more work is needed to better characterise the directionality between cognitive biases and persecutory delusions in schizophrenia.

Studies of gaze processing have also provided evidence for self-relevance biases influencing the perception of gaze direction in schizophrenia. Specifically, substantial evidence has been put forward suggesting that patients are biased to perceive direct gaze when gaze is averted, or in some cases, where there is no physical eye stimulus at all (Hooker and Park, 2005; Rosse et al., 1994; Tso et al., 2012). This body of work also reveals that this bias is likely to reflect a top-down cognitive phenomenon, rather than an aberration of low-level visual processing. Indeed, CFS techniques have revealed that the early visual encoding of faces (Caruana et al., 2019c) and gaze direction (Seymour et al., 2016) are intact in schizophrenia. Patients with schizophrenia have also demonstrated intact processing of gaze direction in conscious viewing tasks that do not involve a self-relevant evaluation of gaze direction (also see Palmer et al., 2018a, Palmer et al., 2018b). Moreover, recent studies have evidenced increased responsivity to communicative gaze cues in patients with schizophrenia (Caruana et al., 2019b). Once again, it has been suggested in the gaze processing literature that these biases may contribute to the experience of persecutory delusions in which the actions of others are perceived as being intentional, communicative and threatening (Abu-Akel and Bailey, 2000; Frith, 2004).

## 5 Conclusion

Paranoid and persecutory delusions (i.e., beliefs that ‘others intend to do me harm’) are a common belief formation aberration experienced in schizophrenia which can cause significant distress for patients (see Appelbaum et al., 1999). The current findings suggest that perhaps in schizophrenia, this may either maintain, or be maintained by, an underlying bias towards the representation of self-directed threat – rather than an increased sensitivity to genuine threat signals. Arguably, the latter is more adaptive for survival as it increases our sensitivity to *genuine* threat signals. A bias towards the perception of threat however, can *potentially* be adaptive when the threat signal is truly present – but maladaptive when this bias leads to the persistent perception of threat in ambiguous or non-threatening situations. Further work is needed to explore this bias in other social information processing paradigms, whilst examining its association with persecutory delusion symptoms in order to verify whether these biases maintain paranoid beliefs, or are shaped by them.

## CRediT authorship contribution statement

**Nathan Caruana:**Conceptualization, Formal analysis, Writing - review & editing, Writing - review & editing.**Christine Inkley:**Investigation, Writing - review & editing.**Marwa El Zein:**Conceptualization, Formal analysis, Writing - review & editing, Writing - review & editing.

## Declaration of competing interest

This research was conducted in the absence of any commercial or financial relationships that could be construed as a potential conflict of interest.

## Appendix A Supplementary data

Additional Model Comparisons. Image 1
