# Supplementary material for: Gaze direction biases emotion categorisation in schizophrenia
Source: Schizophr Res Cogn. 2020 May 21;21:100181. doi: 10.1016/j.scog.2020.100181 (PMC7248285; doi:10.1016/j.scog.2020.100181)
Supplement: Supplementary file 1 — Additional Model Comparisons. [file mmc1.docx]

**Supplementary Material 1 – Additional Model Comparisons**

**Analysis**

We ran an additional, unplanned analysis, which included model comparisons with a third model (i.e., Model 3). This model allowed for an influence of gaze direction on both the sensitivity of the emotions and the decision bias toward choosing one emotion. If model selection revealed Model 3 as the winning model, this would suggest that the increased accuracy for Threat+ faces over Threat- is explained by both (1) an increase in the perceptual recognition for Threat+ faces over Threat- faces, and (2) a bias by gaze direction to categorize emotions which would signal the most threatening cue. As such, the addition of these model comparisons allows us to rule out the possibility that the increased accuracy for Threat+ over Threat- faces may have been explained by an additive, and non-selective, change in the sensitivity and bias parameter.

Model 3 was more complex than Models 1 and 2 because it had an additional free parameter. As such, we accounted for these differences in model complexity by estimating the model evidence using a 10-fold cross-validation estimation of model log-likelihood. This parameter estimation implicitly penalizes for model complexity without relying on particular approximations such as the Bayesian Information Criterion of the Akaike Information Criterion.

**Results**

Consistent with the primary analyses and results, we found that for controls the winning Model 1 (i.e., change in perceptual sensitivity) over Model 2 (i.e., change in the decision bias) still explained the data better than Model 3 (BF_10_ ≈ 10^3.48^, p_exc_ > 0.92). In patients, the winning Model 2 (i.e., change in the decision bias) over Model 1 (i.e., change in perceptual sensitivity) explained the data better than Model 3 (BF_10_ ≈ 10^11.6^, p_exc_ > 0.99). These results further confirm that the effects in each group were specific to a change in sensitivity for controls, and a change in decision bias for patients.
